# Healthcare Workers’ Low Knowledge of Female Genital Schistosomiasis and Proposed Interventions to Prevent, Control, and Manage the Disease in Zanzibar

**DOI:** 10.3389/ijph.2022.1604767

**Published:** 2022-09-15

**Authors:** Humphrey D. Mazigo, Anna Samson, Valencia J. Lambert, Agnes L. Kosia, Deogratias D. Ngoma, Rachel Murphy, Fatma M. Kabole, Dunstan J. Matungwa

**Affiliations:** ^1^ Department of Parasitology and Entomology, Weill Bugando School of Medicine, Catholic University of Health and Allied Sciences, Mwanza, Tanzania; ^2^ Department of Behavioral Sciences, School of Public Health, Catholic University of Health and Allied Sciences, Mwanza, Tanzania; ^3^ Center for Global Health, Weill Cornell Medicine, New York, NY, United States; ^4^ School of Nursing, Catholic University of Health and Allied Sciences, Mwanza, Tanzania; ^5^ Accelerating the Sustainable Control and Elimination of Neglected Tropical Diseases, Crown Agents, London, United Kingdom; ^6^ Crown Agents, London, United Kingdom; ^7^ Ministry of Health Zanzibar, Zanzibar, Tanzania; ^8^ Department of Sexual and Reproductive Health, National Institute for Medical Research, Mwanza, Tanzania; ^9^ Department of Anthropology, School of Arts and Sciences, Rutgers University, New Brunswick, NJ, United States

**Keywords:** healthcare workers, knowledge, female genital schistosomiasis, interventions, qualitative research, Zanzibar

## Abstract

**Objectives:** This study was conducted to explore healthcare workers’ knowledge of female genital schistosomiasis (FGS) and describe proposed interventions to raise awareness about FGS and strengthen healthcare facilities’ capacity to manage FGS cases.

**Methods:** We conducted four cross-sectional focus group discussions and 16 key informant interviews with purposively selected healthcare workers in Zanzibar. Discussions and interviews were digitally recorded, transcribed, and analyzed using NVivo software.

**Results:** Most participants had limited or no knowledge of FGS and lacked skills for managing it. They confused FGS with urogenital schistosomiasis and thought it was sexually transmitted. A few participants knew about FGS and associated it with Human Immunodeficiency Virus (HIV), ectopic pregnancy, cervical cancer, and infertility. To prevent and control FGS, participants proposed interventions targeting communities (including community-based health education) and the healthcare system (including training healthcare workers on FGS).

**Conclusion:** Healthcare workers lacked knowledge of and skills for managing FGS. Besides, healthcare facilities had no diagnostic capacity to manage FGS. Along with on-going interventions to break *S. haematobium* transmission and eventually eliminate urogenital schistosomiasis in Zanzibar, we recommend training healthcare workers on FGS and equip healthcare facilities with medical equipment and supplies for managing FGS.

## Introduction

Female genital schistosomiasis (FGS) is a gynaecological disease caused by *Schistosoma haematobium*, a blood fluke that penetrates the woman’s body when their skin comes into contact with cercariae-infested freshwater [1, 2]. Most women and girls living in endemic areas participate in domestic, recreational, and agricultural activities that involve skin contact with cercariae-infested freshwater. FGS occurs when adult *S. haematobium* eggs are deposited in the cervix and vaginal wall [3]. FGS affects about 20–56 million women and girls worldwide, most of whom live in sub-Sarah Africa [3, 4]. Communities highly endemic for FGS are characterized by low socio-economic status; inadequate water, sanitation, and hygiene services; and limited access to healthcare services [5].

Gynaecological symptoms of FGS include vaginal discharge, abdominal and pelvic pain, dyspareunia (pain with coitus), and post-coital and contact bleeding [6]. Chronic FGS has been associated with such medical conditions as infertility, abortion, ectopic pregnancies, premature birth and low birthweight [6]. Furthermore, there is mounting evidence suggesting that FGS increases the risk of acquiring Human immunodeficiency virus (HIV) [3, 7, 8] and cervical cancer [8].

Zanzibar, a Tanzanian archipelago off the coast of East Africa, used to be highly endemic for *S. haematobium*, a parasite flatworm that causes both urogenital schistosomiasis and FGS [9]. Despite a long history of urogenital schistosomiasis on the Islands dating back to the 1920s [10], school- and community-based mass drug administration (MDA) programs with praziquantel—aiming to lower the prevalence of *S. haematobium*—have been implemented in Zanzibar since the 1980s [11, 12]. Because of these interventions, Zanzibar attained low prevalence levels of *S. haematobium* infections in 2010. Subsequently, the government implemented the Zanzibar Elimination of Schistosomiasis Transmission (ZEST) project from 2012 to 2017 [13, 14] aiming to eliminate urogenital schistosomiasis as a public health problem [15]. The World Health Organization (WHO) defines “elimination as a public health problem” as <1% proportion of heavy intensity infections [16]. ZEST project implemented a combination of bi-annual MDA programs with praziquantel, behavior change activities targeting people’s interactions with cercariae-infested freshwater, and the use of *chemical molluscicides to* target *Bulinus globosus* snails that host *S. haematobium* in Zanzibar [13, 17]. As of 2017, ZEST project had successfully eliminated urogenital schistosomiasis as a public health problem with many parts of Zanzibar attaining <1% proportion of heavy intensity infections. However, the project did not succeed in interrupting the transmission of *S. haematobium* infections, defined as zero incidence of infection [18]. As studies conducted after ZEST project have shown, some areas of Zanzibar have *S. haematobium* infections ranging from low to moderate and high prevalence [18, 19].

The presence and distribution of *S. haematobium*—whether in low or high prevalence—in specific settings in Zanzibar, implies that untreated chronic infections of urogenital schistosomiasis in women and girls can lead to FGS. Despite these indications, FGS has been neglected in both research and policy making circles in Zanzibar. As a result, there is a paucity of research and literature on FGS in Zanzibar [20, 21]. One key step towards addressing FGS in Zanzibar and other parts of sub-Saharan Africa is to have trained healthcare workers and diagnostic equipment to manage (diagnose and treat) this disease [22]. Healthcare workers’ limited or lack of knowledge of FGS and the lack of appropriate diagnostic equipment for managing FGS are likely to lead to underreporting, underdiagnosis, and misdiagnosis of this disease particularly as a sexually transmitted infection (STI) [4].

While broadly aiming to be a conversation starter on FGS in Zanzibar, this study was specifically conducted to explore healthcare workers’ knowledge of FGS and describe proposed interventions to raise awareness about FGS and strengthen healthcare facilities’ capacity to manage FGS cases in Zanzibar. Our study results will complement the strategies for breaking the transmission of urogenital schistosomiasis [19] and help in designing focused interventions to address FGS in Zanzibar.

## Methods

### Study Design and Setting

We conducted a cross-sectional qualitative study using focus group discussions (FGDs) [23] and semi-structured key informant interviews (KIIs) [24]. The study was conducted among healthcare workers working in 14 health facilities (7 from Unguja and 7 from Pemba) located in six (6) *S. haematobium* endemic districts of Zanzibar [25]. Most people in these districts live along and depend on freshwater sources (rivers, marshes, and man-made dams) for domestic and agricultural use.

### Sampling and Recruitment

Using purposive sampling, we recruited 40 healthcare workers to participate in the study (Table 1). The healthcare worker was eligible to participate in the FGD if they were aged ≥18, gave a written and signed informed consent, and consented their views to be audio-recorded. The healthcare worker qualified to be a key informant if they were aged ≥18; were in-charge of the whole facility, department, or unit; gave a written and signed informed consent; and consented their views to be audio-recorded. We set our sample size at four FGDs and 16 KIIs. Prior research indicates that the sample size of two to three FGDs [26] and nine and 24 interviews [27, 28] is sufficient to reach both code and meaning saturation within homogenous groups (Tables 2, 3).

**TABLE 1:** Demographic characteristics of the study participants (Zanzibar, United Republic of Tanzania, 2021).

Variable	Female	Male	Total
Age groups (in years)			
18–40	11	3	14
41+	24	2	26
Sex			
Male	0	5	5
Female	35	0	35
Education level			
Degree	4	1	5
Diploma	22	3	25
Certificate	7	1	8
Secondary	2	0	2
Marital status			
Single	1	0	1
Widow	4	0	4
Married	30	5	35

**TABLE 2:** Number of focus group discussions conducted with healthcare workers (Zanzibar, United Republic of Tanzania, 2021).

FGD code	Participants	Participants’ sex		Place in Zanzibar
		Male	Female	
01	Healthcare workers		6	Unguja Island
02	Healthcare workers		6	
03	Healthcare workers	1	5	Pemba Island
04	Healthcare workers	2	4	
		3	21	Total

**TABLE 3:** Number of key informant interviews conducted with healthcare workers (Zanzibar, United Republic of Tanzania, 2021).

KII code	Participants	Participants’ sex		Place in Zanzibar
		Male	Female	
01	Laboratory Technician		F	Unguja Island
02	Clinical Officer in Charge		F	
03	Nurse Midwife		F	
04	Nurse Midwife		F	
05	Nurse Midwife		F	
06	General Nurse		F	
07	General Nurse		F	
08	General Nurse		F	
09	Registered Nurse		F	Pemba Island
10	Medical Doctor	M		
11	Clinical Officer	M		
12	Clinical Officer		F	
13	Nurse Midwife		F	
14	Nurse Midwife		F	
15	Clinical Officer in Charge		F	
16	Laboratory Technician		F	
		2	14	Total

### Data Collection and Sample Size

Prior to fieldwork, the principal investigator recruited supervisors and research assistants with a track record of conducting qualitative research and gave them a refresher training. After developing the FGD and KII semi-structured topic guides, they were pre-tested by conducting one FGD and two KIIs and then fine-tuned before being used in the actual fieldwork. Data collection was conducted in February 2021. All discussions and interviews took place when participants were off duty to allow them continue serving their patients. FGDs took about one and half to 2 h, while KIIs took about half an hour to 1 h, and 15 min. They were conducted in Kiswahili, led by a moderator, and digitally recorded with the participant’s written and signed consent. A note taker, who also operated a digital audio recorder, captured key issues that participants discussed about FGS. For KIIs, the interviewer audio-recorded the interview and later made brief notes on key issues that emerged during the interview.

### Data Processing and Analysis

After fieldwork, all audio-recorded FGDs and KIIs were transcribed verbatim. FGD and KII transcripts were analyzed as part of a single dataset. The data were analyzed using both deductive and inductive approaches in five steps [29]. First, drawing on the study objectives and the semi-structured topic guides, the study team developed a codebook with initial deductive codes. Second, the initial codebook was enriched by two social scientists who added inductive codes that they identified inductively by reading a few data transcripts. Third, all transcripts were imported on the NVivo software and coded inductively following the enriched codebook. Fourth, codes were developed into more conceptual categories and themes. Finally, relevant quotes were selected—and translated from Kiswahili to English—to demonstrate the findings.

### Ethical Considerations

This study received ethical approvals from the Zanzibar Health Research Ethics Committee (certificate number ZAHREC/03/PR/DEC/2020/29) and Weill Cornell Medicine (certificate number 20-07022381). The study was endorsed by authorities—in the study regions, districts, and shehias—to be conducted in their jurisdictions. The study team adhered to all ethical principles. All participants gave a written consent to participate. Participants’ identities, including their names and names of healthcare facilities where they were working were anonymized. However, since the nature and set up of the focus group compromises guaranteeing full confidentiality and anonymity [30], the study team requested participants to respect the privacy of their colleagues and keep the content of the discussions confidential [30]. For privacy purposes, in this paper, we use code numbers—instead of names—to identify the participants. We have also omitted the names of healthcare facilities for the same purpose.

## Results

We present the study results around two themes. The first theme, healthcare workers’ knowledge of FGS, describes healthcare workers’ knowledge of the aetiology, mode of transmission, symptoms, and treatment of FGS, as well as the association between FGS and other medical conditions. The second theme, interventions to prevent and control FGS, describes respondents’ proposed interventions to prevent and control FGS: interventions targeting the community and the healthcare system.

### Healthcare Workers’ Knowledge of Female Genital Schistosomiasis

Overall, most participants had limited or no knowledge of FGS and held varied misconceptions about this disease. Some, however, made educated guesses about certain aspects of FGS by drawing on their knowledge of urogenital schistosomiasis. Only a few participants had heard of and knew about FGS. Since most participants made reference to urogenital schistosomiasis when discussing about FGS, we have summarized their views as Supplementary Material (see Supplementary File S1).

### Healthcare Workers’ Awareness of Female Genital Schistosomiasis

Most participants had never heard of FGS. Instead, they had heard of urogenital schistosomiasis and some had attended patients suffering from this disease.

I have never heard of FGS. But I have heard of urogenital schistosomiasis many times and have attended patients suffering from this disease. (KII 06).

I have not heard of FGS. I am aware of urogenital schistosomiasis only. But there are few cases of this disease nowadays. Are these diseases the same? (KII 05).

A few participants had heard of FGS but had never seen a patient suffering from this disease.

I have heard other healthcare workers talking about FGS. But I have never seen or attended a patient suffering from this disease [FGS]. (KII 04).

### Aetiology and Mode of Transmission of Female Genital Schistosomiasis

Despite being unaware of FGS, most participants made educated guesses and named *Schistosoma* parasites as its aetiological agent. However, these participants did not know how urogenital schistosomiasis is associated with FGS. Their responses about the aetiology of FGS drew on their knowledge and experience of urogenital schistosomiasis. Some participants explained that, like urogenital schistosomiasis, FGS is transmitted when a person’s skin comes into contact with cercariae-infested freshwater when engaging in such activities as farming, swimming, bathing, and washing clothes and dishes. In one of the discussions, one participant with excellent knowledge of FGS succinctly described its aetiology, development, symptoms, and complications.

In women, when Schistosoma parasite eggs lodge in the genital track, they cause damage [and lead to such] symptoms as blood in urine, vaginal discharge, and abdominal and pelvic pain. This disease is called FGS. If FGS is not treated, a woman can become infertile. In a pregnant woman, it can lead to ectopic pregnancy. (FGD 01).

A few participants did not know the aetiology of FGS although they knew the aetiology of urogenital schistosomiasis. Other participants confused aetiology with mode of transmission of FGS. They also had misconceptions about its mode of transmission. The key misconception they had was that FGS can be transmitted through sexual activity.

When a man suffering from FGS has sexual intercourse with an uninfected woman, she can also catch FGS. The man will ejaculate sperms containing FGS parasites into the woman’s genitalia. The parasites will infect the woman’s reproductive system and develop into FGS. (FGD 02).

However, some participants in the same FGD, who had knowledge of how FGS is transmitted, addressed this misconception and explained that FGS is transmitted when a person’s skin comes into contact with cercariae-infested freshwater and not otherwise.

Schistosomes are not sexually transmitted. They live in certain freshwater snails. Their infectious form, called cercariae, are released into the water. Cercariae penetrate the human skin when a person’s skin comes into contact with freshwater contaminated with cercariae. Cercariae exit the human body only when the host [human being] defecates or urinates. (FGD 02).

### Symptoms of Female Genital Schistosomiasis

Most participants did not know the symptoms of FGS. Instead, while drawing on their knowledge of urogenital schistosomiasis, they made educated guesses about the symptoms of FGS. However, a few participants had excellent knowledge of FGS, particularly its symptoms, including haematuria (blood in urine), abdominal and pelvic pain, dyspareunia, vaginal discharge, and dysuria (painful urination). When asked about what symptoms a woman or a girl suffering from FGS shows, participants responded that:

A woman or girl suffering from FGS will experience extreme abdominal pain. She will also feel pain during sexual intercourse. (KII 02).

Some of the symptoms of FGS are abdominal and pelvic pain and blood in urine. Sometimes a woman can experience vaginal discharge with a very unpleasant odor. (KII 10).

Some women [suffering from FGS] may feel pain during sex. They also experience uncomfortable, painful, and sometimes burning urination. (KII 12).

### Association Between Female Genital Schistosomiasis and Other Medical Conditions

Although most participants did not know how FGS is associated with other medical conditions, when left untreated, FGS leads to complications including infertility, ectopic pregnancy, and genital ulcers. It also heightens the risk of HIV and Human papillomavirus (HPV) infections. A few participants with knowledge of FGS explained how it is associated with other medical conditions.

There was a woman who visited our healthcare facility on multiple occasions complaining about infertility. When the doctor diagnosed her, they found that she had FGS. Her infertility was a result of untreated FGS. (FGD 03).

Women suffering from FGS are at risk of other diseases. For instance, when schistosomes damage the patient’s reproductive organs, they increase their risk of HIV and HPV infections. (FGD 04).

Women suffering from FGS have a higher risk of HIV and HPV infection than other women. Also, pregnant women suffering from FGS are more likely to have an ectopic pregnancy. (KII 10).

### Treatment of Female Genital Schistosomiasis

Most participants did not know the treatment for FGS. Only a few (all medical doctors, *n* = 5) knew that praziquantel is the drug used to treat FGS. However, all participants knew that praziquantel is used to treat urogenital schistosomiasis because they had participated in implementing community- and school-based MDA programs in Zanzibar. Since most of them did not know the relationship between FGS and urogenital schistosomiasis, they could not tell if praziquantel is used to treat both diseases.

### Proposed Interventions to Mitigate Female Genital Schistosomiasis

We asked the participants to propose interventions suitable for raising awareness of FGS among healthcare workers and strengthening healthcare facility case management capacity. Broadly, participants proposed interventions targeting communities and the healthcare system.

### Interventions Targeting Communities

Participants proposed two forms of interventions targeting communities. First, they proposed community-based health education to raise people’s awareness and knowledge of the aetiology, symptoms and related complications, mode of transmission, groups of people at risk, and treatment of FGS. This knowledge will reduce the stigmatizing attitudes that people have against women and girls suffering from FGS. More importantly, community-based health education will improve people’s treatment-seeking behavior to seek health services from healthcare facilities. Community-based health education could be delivered to the community members by healthcare workers or community health workers.

Community-based health education is an important intervention for helping people to learn about FGS including where to seek treatment [at the healthcare facilities]. It could be delivered by healthcare workers or community health workers. (FGD 01).

Second, participants proposed that since the transmission of FGS is through skin contact with cercariae-infested freshwater, there is need for the government to improve and invest more in water supply, sanitation, and hygiene (WASH). If the improvement and investment in WASH are done simultaneously with other interventions such as community- and school-based MDA programs and improvement of the healthcare system (next subsection), they could enhance the prevention of schistosomiasis including FGS.

The government should ensure that safe water is available and accessible to all people in the communities [rural and urban]. If running water is available and accessible to all, less people will use infested water from rivers or ponds. If this is realized, the transmission of schistosomes will be reduced significantly. (KII 10).

### Interventions Targeting the Healthcare System

Participants proposed three forms of interventions for improving the healthcare system’s capacity to manage FGS. First, they proposed that the government and other stakeholders should organize and fund trainings aiming to improve healthcare workers’ knowledge of FGS.

The government and other stakeholders can organize and fund trainings on FGS for all healthcare workers. When healthcare workers have knowledge of FGS, they will be in a good position to manage it. (FGD 03).

Second, participants proposed that the government and other stakeholders should work together to ensure the healthcare facilities have medical equipment and supplies for managing FGS. The government must also address the long-standing problem of understaffing by recruiting healthcare workers required at different levels of the healthcare system.

If the government purchases enough medical equipment and supplies on time, our health care facilities will have the capacity to manage cases of FGS and other diseases. When this is done, plus community health education, the number of patients seeking for treatment services at the healthcare facilities will increase. The government should also solve the problem of understaffing. If the number of staff is increased and all other things are in good order, then we can offer high quality healthcare services to our patients. (FGD 02).

Third, participants proposed integrating FGS, HIV/AIDS, other STIs, and HPV/cervical cancer screening services since most of the symptoms of these diseases are similar. Thus, when a patient presents to the healthcare facility with the symptoms of any of these diseases, they should be screened for all of them. Integration of these services enables the management of cases of different medical conditions at one point.

If a patient presents at the healthcare facility with a symptom of schistosomiasis, they must also be screened for HIV, other STIs, and cervical cancer as some of the symptoms of these medical conditions are similar. This approach will help us manage more cases of diseases with similar symptoms. (KII 10).

## Discussion

Our findings demonstrate that most healthcare workers had limited or no knowledge of FGS across seven aspects: awareness, aetiology, mode of transmission, symptoms, association with other medical conditions, and treatment. While some healthcare workers demonstrated that they knew about FGS, we learned that their responses were educated guesses based on their knowledge and experience of managing urogenital schistosomiasis. Only a few healthcare workers—all of whom were medical doctors (*n* = 5)—demonstrated excellent knowledge of FGS.

Most participants held misconceptions that FGS can be transmitted through sexual activity and that it is itself an STI. Similar findings were reported by a study conducted in Ghana [31]. This finding was worrying because healthcare workers’ limited or lack of knowledge of and misconceptions about FGS are likely to lead to misdiagnosis and referring patients to a wrong specialist or other treatment services. The intersection of all these situations can lead to serious consequences on women and girls’ reproductive health. As one study conducted in Ghana reported, healthcare workers with limited or no knowledge of FGS were more likely to refer women and girls—presenting with symptoms of FGS—to the STI clinic [31]. Furthermore, misdiagnosing FGS as an STI may negatively influence treatment-seeking behavior particularly among women and girls with symptoms of FGS. For instance, a study conducted in Tanzania reported that women with symptoms of FGS were reluctant to seek treatment from the healthcare facilities because they feared being stigmatized and mistreated if they were diagnosed with an STI [32].

One serious setback in addressing FGS in areas where *S. haematobium* is endemic is the lack of knowledge of and skills to manage FGS among healthcare workers [3]. Although most parts of Zanzibar have low prevalences of *S. haematobium* [18, 19], there are still a few areas with “consistent or recurring moderate or high prevalence” of this parasite [19]. With interventions targeting these hotspots already proposed [19], emphasizing on increasing knowledge of FGS among healthcare workers (and other stakeholders such as community members) will supplement the efforts for interrupting the transmission of *S. haematobium* in Zanzibar.

Participants proposed a number of interventions to resolve the challenges surrounding the transmission of *S. haematobium* and FGS in Zanzibar. These challenges include community and healthcare workers’ limited or lack of knowledge of FGS, healthcare workers’ inadequate skills to manage FGS, and healthcare facilities’ inadequate capacity to manage FGS mainly because of the lack of diagnostic medical equipment such as microscopes and gynecologic specula. Participants proposed interventions targeting the community and ones targeting the healthcare system. They proposed community-based health education as a suitable intervention for improving both community members’ knowledge of FGS as well as women and girls’ treatment-seeking behavior for FGS. Available evidence suggests that community-based health education interventions are effective in improving community members’ health-seeking behavior [33, 34]. Furthermore, participants proposed that the government needs to invest and improve the supply of and access to safe and clean water to mitigate the transmission of *S. haematobium,* which happens mainly through human skin contact with cercariae-infested freshwater. Previous studies have shown that improving and investing in access to safe and clean water, adequate sanitation, and hygiene significantly reduce the transmission of *S. haematobium* [35].

To improve healthcare workers’ knowledge of and skills for managing FGS, participants proposed offering them in-service training. Research has shown that in-service, competence-based trainings improve healthcare workers’ knowledge and skills for managing various diseases [36]. Participants also proposed that the Tanzanian healthcare system must integrate FGS, HIV and other STIs, and cervical cancer screening services [8] and offer them under the umbrella of “SRH services.” However, this intervention can only be productive if competent and skilled healthcare workers are available to deliver these services [8, 22]. Finally, participants proposed that the government and other stakeholders need to work together to equip healthcare facilities with diagnostic medical equipment such as microscopes and gynecologic specula. Inadequate capacity of healthcare facilities to manage FGS is a common observation in most schistosomiasis-endemic areas [37–39].

### Limitations

Despite the strengths of our findings, our study has three limitations. First, the study had more female (*n* = 35) than male (*n* = 5) participants. However, this was expected because Zanzibar’s healthcare force is generally dominated by women. Nursing is the most gender-skewed cadre. For instance, there were 1,042 female nurses in 2017 compared to 271 male nurses [40]. Second, we could not verify our participants’ responses and perceived experiences on FGS as these were reported responses. But generally, FGS was a new topic to most participants. Third, this study focused on FGS among women and girls. No question was asked about male genital schistosomiasis (MGS). However, as studies have reported, in areas where urogenital schistosomiasis is endemic, MGS is a potential public health problem. This could be the case in Zanzibar. Despite these limitations, our findings demonstrate that healthcare workers’ limited or lack of knowledge of FGS and lack of diagnostic capacity for managing FGS in the health facilities are the two challenges that require immediate attention if FGS is to be addressed and *S. haematobium* is fully eliminated in Zanzibar.

### Conclusion

Most healthcare workers had limited or no knowledge of FGS and lacked skills for managing it. Healthcare facilities also lacked diagnostic capacity to manage FGS. We recommend offering in-service, competence-based training to healthcare workers to improve their knowledge of and skills for managing FGS. We also recommend equipping the healthcare facilities with diagnostic equipment for managing FGS. However, we recognize that it may take time before all health facilities are fully equipped with all required FGS diagnostic equipment. We therefore propose developing a simple diagnostic algorithm—which combines demographic information, history of exposure to risk environment, and symptoms of FGS—to help healthcare workers screen, identify, and refer FGS cases to the healthcare facilities that have the capacity to manage this disease. Finally, since FGS, HIV, and cervical cancer have serious sexual and reproductive health and rights consequences for women and girls, we follow the footsteps of other researchers [8] to recommend integrating prevention and control measures of these diseases.

## Data Availability

The data presented in this study are available on request from the Directorate of Research and Publications of the Catholic University of Health and Allied Sciences (Email: vc@bugando.ac.tz). The data are not publicly available due to the fact that they contain information that can reveal the identities of the subjects.

## Supplementary Material

Supplementary Data Sheet S1 FGD Guide.

Supplementary Data Sheet S2 KII Guide.

Supplementary Data Sheet S3 Codebook.

Supplementary File S1 Knowledge of urogenital schistosomiasis.
