# Supplementary material for: Healthcare Workers’ Low Knowledge of Female Genital Schistosomiasis and Proposed Interventions to Prevent, Control, and Manage the Disease in Zanzibar
Source: Int J Public Health. 2022 Sep 15;67:1604767. doi: 10.3389/ijph.2022.1604767 (PMC9520356; doi:10.3389/ijph.2022.1604767)
Supplement: Supplementary file 1 [file Table1.docx]

**Supplementary File 1**

**Table 4: Healthcare workers’ awareness and knowledge of urogenital schistosomiasis (Zanzibar, United Republic of Tanzania, 2021)**

| **Themes** | **Summary of the participants’ views** | **Illustrative Quotations** |
| --- | --- | --- |
| Awareness of urogenital schistosomiasis | Most participants were aware of urogenital schistosomiasis because of the interventions that were implemented in communities and schools for a long time. | - I have known about schistosomiasis since I was young, when I was still in school. In the past, healthcare workers used to coming in the communities and schools to diagnose schistosomiasis. Some students would be diagnosed with schistosomiasis. These students had such symptoms as blood in urine and pain when urinating. Once diagnosed with schistosomiasis, the healthcare workers would give them medication. (FGD 01). |
|  | Some participants knew urogenital schistosomiasis because they manage cases of this disease. | - I have heard of urogenital schistosomiasis many times. I know it very well because I have tested many samples of people who suffer from this disease. (KII 09). |
| Symptoms of urogenital schistosomiasis | Most participants were aware of the common symptoms of urogenital schistosomiasis: blood in urine (haematuria), abdominal and pelvic pain, and pain when urinating (dysuria). | - It is a terrible disease. Some of its common symptoms are blood in urine, pain and burning when urinating, and abdominal pain. Patients may also experience pelvic pain. (KII 15). |
| Body parts affected by urogenital schistosomiasis | Participants mentioned most body parts that are affected by urogenital schistosomiasis as being located in the urinary and gastrointestinal systems. | - Urogenital schistosomiasis affects the abdomen and the reproductive system including the genitals. (KII 01). - Schistosomiasis affect the urinary system and gastrointestinal system. When the urinary system is affected, a person gets urogenital schistosomiasis. If the gastrointestinal system is affected a person gets intestinal schistosomiasis. (KII 10). - Urogenital schistosomiasis affects mainly the pelvic organs such the cervix, uterus, bladder, urethra and vagina. (KII 16). |
| Aetiology of urogenital schistosomiasis | Most participants correctly explained that Schistosoma parasites cause urogenital schistosomiasis. | - Urogenital schistosomiasis is a disease that affects the urinary system. It is caused by small parasites called Schistosoma. (KII 04). |
| Modes of transmission of urogenital schistosomiasis | Most participants correctly explained how urogenital schistosomiasis is transmitted, that is, transmission occurs when uninfected people make skin contact with freshwater that has been contaminated with faeces or urine from the person suffering from urogenital schistosomiasis. | - When a person with schistosomiasis defecates or urinates in a water source and then an uninfected person steps in the same water, they will be infected. Also, people who will wash clothes or bath in this water will be infected. (KII 15). - If a person suffering from urogenital schistosomiasis urinates in the water, and you step in that water [your skin comes into contact with that water], you can also get infected [with Schistosoma parasites]. (FGD 01). |
|  | Skin contact with contaminated freshwater may occur when engaging in such activities as swimming, bathing, washing clothes, or playing in pond or river water. | - When people go to the rivers for such activities as swimming, if there are snails which carry Schistosoma parasites, all who will be swimming may be infected [by those parasites]. (FGD 01). - When children play or swim in pond or river water, they may be infected with parasites that cause urogenital schistosomiasis. (FGD 02). - People who wash clothes or bath in contaminated freshwater may be infected with [Schistosoma parasites]. (KII 15). |
| Perceived prevalence of urogenital schistosomiasis | Most participants perceived that the infection rate of schistosomiasis was very low. | - The prevalence of urogenital schistosomiasis is very low. And I have not heard or seen a patient who has died of urogenital schistosomiasis. (KII 15). - We can get two or three patients with urogenital schistosomiasis in one month. And we do not get any case in some months. (FGD 01). |
|  | A few participants observed that the prevalence of urogenital schistosomiasis has declined mainly because of school- and community-based MDA programs. | - There are school- and community-based intervention programs delivering treatment [praziquantel] to control urogenital schistosomiasis. That is why the prevalence of urogenital schistosomiasis has declined. But the disease has not been fully controlled. I have witnessed people in the communities and hospitals being diagnosed with this disease. (FGD 03). |
| Groups of people at high risk of urogenital schistosomiasis | Participants observed that all groups of people are vulnerable to urogenital schistosomiasis. However, women and boys have a heightened risk of being infected with parasites that can cause urogenital schistosomiasis. | - Women and men go to the river to wash their clothes. Women wash the dishes and bath their children at the rivers. Children swim and play in the rivers. Thus, people of all age groups are vulnerable to this disease [urogenital schistosomiasis]. (FGD 03). - All people are at risk. Their risk can differ, but they are all vulnerable. As long as we still have the parasites in freshwater sources such as rivers and ponds, and our people still use these as sources of water for domestic or recreational activities, all groups of people who interact with these water sources are at risk of being infected with Schistosoma [parasites that cause urogenital schistosomiasis]. (KII 10). |
|  | Women are at risk because of doing domestic chores which involve skin contact with contaminated water. | - Women, particularly those who live along the rivers, are at high risk of urogenital schistosomiasis. They will wash clothes and bath their children at the river because they want to save money on their water bills or because of water scarcity [no running water in their homes]. (FGD 02). |
|  | Boys are at risk because they engage in recreational activities such as swimming in rivers and ponds. | - In our communities, boys have more freedom than girls. They are more outgoing because than girls. So, they are free to play anywhere. They swim in the rivers and ponds without knowing that they can be infected with those parasites [that can cause urogenital schistosomiasis. (FGD 04). |
|  | Girls have a low risk because they engage in domestic chores and are less outgoing. | - They are also vulnerable [to urogenital schistosomiasis]. But not like boys. They are always at home attending domestic chores: washing dishes, washing clothes, cooking, and cleaning homes. But they also go to fetch water at rivers. So, they can also be infected [with parasites that cause urogenital schistosomiasis. (FGD 03). |
| Treatment of urogenital schistosomiasis | All participants knew that urogenital schistosomiasis is treated by a prescription drug Praziquantel | - Urogenital schistosomiasis is treated using drug Praziquantel. (FGD 02). |
