# Supplementary material for: Healthcare Workers’ Low Knowledge of Female Genital Schistosomiasis and Proposed Interventions to Prevent, Control, and Manage the Disease in Zanzibar
Source: Int J Public Health. 2022 Sep 15;67:1604767. doi: 10.3389/ijph.2022.1604767 (PMC9520356; doi:10.3389/ijph.2022.1604767)
Supplement: Supplementary file 2 [file DataSheet3.docx]

**CODEBOOK**

| **Name** | **Description** |
| --- | --- |
| Associating FGS with other diseases or conditions | All information indicating how people associate FGS with other infections/diseases. |
| Cervical cancer | All information on how people associate FGS with cervical cancer and the reasons for this association. |
| Ectopic pregnancy | All information on how people associate FGS with Ectopic pregnancy (or Extrauterine pregnancy) and the reasons for this association. |
| HIV or AIDS | All information on how people associate FGS with HIV/AIDS and reasons behind this association. |
| Infertility | All information on how people associate FGS with infertility and the reasons for this association. |
| Miscarriage | All information on how people associate FGS with miscarriage and the reasons for this association. |
| Other STIs | All information on how people associate FGS with other STIs such as Gonorrhea and Syphilis and the reasons for this association. |
| Availability of FGS services | All information on the availability of health services for FGS including equipment, health service providers, and incentives for health services providers. |
| Health facilities do not have enough health workers to fight against FGS | All information indicating that health facilities do not have enough health workers to fight against FGS. |
| Health facilities do not have incentives to motivate health workers | All information indicating that health facilities do not have enough incentives to motivate health workers in the fight against FGS. |
| Health facilities have enough equipment to diagnose FGS | All information indicating that health facilities have enough equipment to diagnose FGS. |
| Health facilities have enough health workers to fighting against FGS | All information indicating that health facilities have enough health workers to fight against FGS. |
| Health facilities have fewer or no equipment to diagnose FGS | All information indicating that health facilities do not have enough or have no equipment to diagnose FGS. |
| Health facilities have incentives to motivate health workers | All information indicating that health facilities have enough incentives to motivate health workers in the fight against FGS. |
| Awareness of FGS | All information indicating that study participants are aware or not aware of FGS. |
| Heard about FGS | All information indicating that people have ever heard or never heard about FGS. |
| Never heard about FGS | All information indicating that people have never heard about FGS. |
| Awareness of urogenital schistosomiasis | All information about people’s knowledge or perception of schistosomiasis. |
| From whom people have heard about urogenital schistosomiasis | Human sources of information about urogenital schistosomiasis e.g. friends, health workers etc. |
| Heard about urogenital schistosomiasis | Information indicating that people have ever heard about urogenital schistosomiasis. |
| Local terms of urogenital schistosomiasis | Local phrases/words for urogenital schistosomiasis. |
| Never heard about urogenital schistosomiasis | Information signifying that people have never heard about urogenital schistosomiasis. |
| Prevalence of urogenital schistosomiasis | All information that describes the prevalence of urogenital schistosomiasis in a given village community. |
| Severity of urogenital schistosomiasis | All information that describes the severity of urogenital schistosomiasis in a given village community. |
| Where people have heard about urogenital schistosomiasis | Non-human sources of information about urogenital schistosomiasis e.g. Newspapers, radio or TV programs, leaflets, photographs, health facilities etc. |
| Causes/Aetiology of FGS | All statements stating the causes of FGS. |
| FGS treatment seeking behavior | Places/treatment facilities where people/women go when they find out that they have symptoms of FGS. All information describing the first step a woman who has symptoms of FGS will take and the reasons for taking such a step. |
| Biomedical health facilities | All information explaining that people visit biomedical health facilities—e.g. health centres and hospitals—to seek for the treatment of FGS. Also information about what treatment people get at the health facilities. |
| Private retail drug shops | All information explaining that people visit private retail drug shops to seek for the treatment of FGS. Also information about what treatment people get from the private retail drug shops. |
| Self-treatment | All information explaining that people resort to self-treatment using herbal medication when they find out that they are infected with FGS. Also information about what people treat themselves with and how. |
| Traditional healers | All information explaining that people visit traditional healers to seek for the treatment of FGS. Also information about what treatment people get from traditional healers. |
| Health workers ever received FGS training | Information about whether health workers have ever received FGS training |
| Funder of the training | The person or institution that funded the training. |
| When | When they received the training. |
| Human body parts affected by urogenital schistosomiasis | All statements in which study participants describe parts of the human body that are affected by urogenital schistosomiasis. Also, any explanations on how those parts of the body are affected. |
| Interventions | All interventions, programs, or strategies suggested to prevent, control, and manage FGS. They can be community-based (targeting community members) and/or facility based (targeting the health system including healthcare workers) |
| Meaning of urogenital schistosomiasis | All information in which study participants define urogenital schistosomiasis or explain the meaning of urogenital schistosomiasis. |
| Men’s reaction to FGS infected partners | All information that describes how men would react if they found out that their partners/wives are infected with FGS. |
| Most at risk population for urogenital schistosomiasis | All groups of people mentioned by study participants as being at high risk for urogenital schistosomiasis. |
| Children and why children are at risk | All statements stating that children are at high risk for urogenital schistosomiasis and specifying their age. Also, why they are at risk |
| Men and Why men are at higher risk | All statements stating that men are at high risk for urogenital schistosomiasis. Also, why they are at risk |
| Women and why women are at higher risk | All statements stating that women are at high risk for urogenital schistosomiasis. Also, why women are at higher risk |
| Prevalence of FGS | All information describing how big/small is the problem of FGS compared to other health problems in the village communities. |
| Symptoms of FGS | All features mentioned by study participants indicating that a woman is infected by FGS. Such features may be or may not be apparent to a patient. |
| Symptoms of urogenital schistosomiasis | All information/features mentioned by study participants indicating that a person is infected by urogenital schistosomiasis. Such features may be or may not be apparent to a patient. |
| Transmission of FGS | All information about the transmission of FGS from an infected woman to another person, be a man, woman, or children. |
| FGS can be transmitted to a sexual partner | All information about whether or not a woman/girl infected with FGS can infect her husband/sexual partner. Also, all information describing WHY study participants think this can or cannot happen. |
| FGS can be transmitted to another person | All information where study participants acknowledge that FGS can be transmitted from an infected woman to another person. Also, all information describing how such transmission occurs. |
| FGS cannot be transmitted to another person | All information where study participants mention that FGS cannot be transmitted from an infected woman to another person. Also, all information describing why FGS cannot be transmitted from an infected woman to another person. |
| Transmission of urogenital schistosomiasis | All statements in which study participants explain how urogenital schistosomiasis is transmitted 1) from physical sources to the people and 2) from one person to another. In the second case, transmission may involve certain human behaviors and practices that shape the transmission of urogenital schistosomiasis. |
| Treatment of urogenital schistosomiasis | Places/treatment facilities where people/women go when they find out that they have symptoms of Urogenital schistosomiasis. All information describing the first step a woman who has symptoms of urogenital schistosomiasis will take and the reasons for taking such a step. |
