# Supplementary material for: Healthcare Workers’ Low Knowledge of Female Genital Schistosomiasis and Proposed Interventions to Prevent, Control, and Manage the Disease in Zanzibar
Source: Int J Public Health. 2022 Sep 15;67:1604767. doi: 10.3389/ijph.2022.1604767 (PMC9520356; doi:10.3389/ijph.2022.1604767)
Supplement: Supplementary file 4 [file DataSheet1.docx]

**FOCUS GROUP DISCUSSION GUIDE**

**Other Healthcare workers (HCWs) – not serving as in-charge of the healthcare facility, department, or unit**

**Introduction**

Thank you for accepting to participate in this discussion. As healthcare workers, we value your knowledge and experience about urogenital schistosomiasis and Female genital schistosomiasis. We are therefore holding this discussion with you to get your perspective. Data that will be generated from this discussion will help us in suggesting interventions to prevent and control the transmission of Female Genital Schistosomiasis (FGS) in Zanzibar.

**Discussion rules**

- Every participant will air their views without fear of any other participant.
- Giving each other time to speak. When one participant is speaking, others will be listening.
- Respecting the opinions/views of others. We disagree on certain issues, but no one should ridicule the other.
- There is no right or wrong response. All opinions/views are equally important.

**I. Urogenital Schistosomiasis**

1. What are the health problems facing your community? Please, mention them.

For each of the health problem mention, probe the following:

- Prevalence?
- Severity?
- Disability?
- Morbidity?

1. Have you ever heard about urogenital schistosomiasis? How is it called in your local language?

- Where did you hear it?
- From who did you hear?
  - What is the prevalence?
  - What is the severity of the problem?

1. What is urogenital schistosomiasis in your opinion?
2. What are the causes of urogenital schistosomiasis? (The sources of urogenital schistosomiasis)
3. How is urogenital schistosomiasis transmitted from one person to another?

Probe

- Behaviours that contribute to the transmission of urogenital schistosomiasis
  - Defecation in the water sources
  - Not using toilets/latrines (Open defecation)
  - Rice farming (in paddy fields)
  - Swimming in the water sources

1. In your opinion, what are the symptoms of urogenital schistosomiasis
2. What parts of the human body are affected by urogenital schistosomiasis?
3. Which groups of people in your community are most affected by urogenital schistosomiasis?

- Children. Of which age? How are they affected?
- Men. Of which age? How are they affected?
- Women. Of which age? How are they affected?

**II. Female Genital Schistosomiasis**

1. Have you ever heard about Female Genital Schistosomiasis?

Probe: If they have never heard about it, remind them what they have said in the previous question (in case they indicated that they have heard about it).

If they respond that they have heard about it:

1. What are the causes of Female Genital Schistosomiasis?
2. What symptoms does a woman who is infected with Female Genital Schistosomiasis display? Mention them.

Probe

- Blood in urine (haematuria)
- Abdominal and pelvic pain
- Increased vaginal discharge
- Pain with coitus (Dyspareunia)
- Post-coital bleeding
- Menstrual disorders
- Dysuria (pain or difficulty urinating)
- Genital lesions

1. Can a woman with Female Genital Schistosomiasis transmit it to another person? Je,
   1. If yes, how?
   2. If no, why?
2. In your view, can a woman/girl infected with Female Genital Schistosomiasis infect her husband/sexual partner?
3. Which groups of women are at more risk of being infected with Female Genital Schistosomiasis?

Probe

- Older women. Why?
- Women in their reproductive age. Why?
- Girls. Why?

1. In your opinion, how do people associate Female Genital Schistosomiasis with other infections/diseases?

Probe: How do people associate Female genital Schistosomiasis with?

- HIV and AIDS
- Other sexual transmitted infections (e.g. [Gonorrhea](https://www.cdc.gov/std/gonorrhea/default.htm), [Syphilis](https://www.cdc.gov/std/syphilis/default.htm))
- Cervical cancer
- Ectopic pregnancy (or Extrauterine pregnancy)
- Miscarriage
- Infertility/sterility
- Other problems associated with fertility and pregnancy

1. In your opinion, how does the community perceive women/girls infected with Female Genital Schistosomiasis?

Probe

- How does the community regard/perceive a girl infected with Female Genital Schistosomiasis?
- How does the community regard/perceive an older woman infected with Female Genital Schistosomiasis?
- How does the community regard/perceive a married woman infected with Female Genital Schistosomiasis?
- Is there any kind of stigmatization against women/girls infected with Female Genital Schistosomiasis in this community?
  - Can you describe what form of stigmatization that is?
- Is there any kind of stigmatization against women/girls infected with sexually transmitted infections (e.g. HIV/AIDS, [Gonorrhea](https://www.cdc.gov/std/gonorrhea/default.htm), and [Syphilis](https://www.cdc.gov/std/syphilis/default.htm)) in this community? Female Genital Schistosomiasis in this community?
  - Can you describe what form of stigmatization that is?

1. In your opinion, in order to reduce/decrease stigmatization against women infected with Female Genital Schistosomiasis

- What should the society/community do, considering that you are also members of the society/community?
- What should the government and other institutions do in this community?
- What should the government and other institutions do in the health sector?

1. In your opinion, what should be done in your community to encourage women and girls to seek for Female Genital Schistosomiasis health services?

Probe

- What should be done to encourage women to access health services for HIV/AIDS and other sexually transmitted infections ([Gonorrhea](https://www.cdc.gov/std/gonorrhea/default.htm) and [Syphilis](https://www.cdc.gov/std/syphilis/default.htm))?

1. **Health services and treatment of Female Genital Schistosomiasis**
2. Tell the group: Think of a woman you know in your community (she can be a girl, sister, nephew, friend etc.) has some of the symptoms of Female Genital Schistosomiasis (for instance has blood in urine). Let as call her……..(Put a name). What is the first step that she will take? Why?

- Do health workers here have enough knowledge on Female genital Schistosomiasis?
- Can health workers know if the symptoms she displays are those of Female genital Schistosomiasis?
- Are there other infections/diseases that the health workers would think of first or prioritize instead of Female Genital Schistosomiasis?
- What are the health services available for Female Genital Schistosomiasis treatment?
- Do you think that health facilities have enough equipment to diagnose patients infected with Female Genital Schistosomiasis?
- If not, why?
- Probe: the availability of the medical equipment, health workers, incentives etc.

1. What are the challenges that face
2. Health workers providing services to women infected with Female Genital Schistosomiasis?
3. Health workers providing services to women infected with other sexually transmitted infections?
4. **Interventions to prevent and control Female Genital Schistosomiasis**

| Duration | 30 minutes |
| --- | --- |
| Moderator’s guide | Write the question on the flipchart  Draw a table on the flipchart for the second question for every group   \| Types of interventions \| Why this intervention? \| Who should deliver the intervention? \| Anticipated challenges in implementing the intervention \| \| --- \| --- \| --- \| --- \| \|  \|  \|  \|  \| |

1. Create groups: Request the participants to seat in pairs and discuss the following question:
   - What do you think should be done so that women and girls could get treatment services for Female Genital Schistosomiasis?

N.B. Ask every group to share their opinions/views and write them on the flipchart.

1. Small groups: Request groups to join other groups and discuss:

- If the government or a non-governmental organization wants to support your community to fight against Female Genital Schistosomiasis, what kind of interventions could work (in this community?
- Why do you think these interventions could work better?
- Who do you think should deliver these interventions?
- Are there challenges in implementing these interventions?

1. If the government or a non-governmental organization implements a community-based teaching intervention against Female Genital Schistosomiasis in your community (on raising awareness about Schistosomiasis, treatment and prevention of Schistosomiasis as well as women/girls’ seeking behaviour:

- Do you think this community-based teaching intervention can have positive impacts by improving women/girls’ awareness on health seeking behaviour and accessing treatment for Female Genital Schistosomiasis? If yes, why? If no, why?
- If yes, how should this community-based teaching intervention be run?
- Probe: Topics to be discussed, venue, teaching materials/equipment, time for the training, who should deliver the intervention etc.
- Do you think it is important to involve men in this community-based teaching intervention against Female Genital Schistosomiasis? If yes, why? If no, why?

1. In your opinion, do you think there will be any challenges in implementing this intervention?

- If yes, what are those challenges?
- In your view, how should those challenges be resolved?

1. Have you ever been trained on diagnosing Female Genital Schistosomiasis? If yes, when, where, who delivered the training (facilitator), and who funded the training?

**The end**

We have come to the end of our discussion. Do you have anything regarding the topic we have discussed that you would want to share with us? Or is there anyone with a question?

Thank you so much for your time and responses.
